# Extraction Kinetics of Phenolic Antioxidants from the Hydro Distillation Residues of Rosemary and Effect of Pretreatment and Extraction Parameters

**DOI:** 10.3390/molecules25194520

**Published:** 2020-10-02

**Authors:** Irini Psarrou, Antigoni Oreopoulou, Dimitrios Tsimogiannis, Vassiliki Oreopoulou

**Affiliations:** 1 Laboratory of Food Chemistry and Technology, Department of Chemical Engineering, National Technical University of Athens, 5 Iroon Polytechniou, Zografou, 15780 Athens, Greece; irenepsarrou120@gmail.com (I.P.); antigoni@vioryl.gr (A.O.); ditsimog@chemeng.ntua.gr (D.T.); 2 Vioryl, Agricultural and Chemical Industry, Research S.A., 28th km National Road Athens-Lamia, Afidnes, 19014 Attiki, Greece; 3 NFA (Natural Food Additives), Laboratory of Natural Extracts Development, 6 Dios st, Tavros, 17778 Athens, Greece

**Keywords:** rosemary, solvent extraction, kinetics, phenolics, UAE

## Abstract

Rosemary residue, remaining after the distillation of essential oil, is currently unexploited, while it is a source of phenolic antioxidant components. This raw material was used for the extraction of phenolic compounds by aqueous ethanol or acetone in a continuously stirred reactor. The experimental results were fitted with a two-stage diffusion model. The highest extraction rates, total phenolic content (TPC) recovery, and 2,2-diphenyl-1-picrylhydrazyl (DPPH) radical scavenging capacity were obtained by acetone 60% and ethanol 60%. Grinding of the raw material enhanced the extraction rate and increased TPC yield and antioxidant capacity as the particle size decreased. Pre-treatment by maceration in water (4 h) dissolved a high amount of TPC and shortened the extraction time, while the combination with the pulsed electric field process did not provide further improvement. The use of ultrasound increased the efficiency of the extraction.

## 1. Introduction

Rosemary is the most well-known aromatic plant with antioxidant activity. Several researchers have reported radical scavenging and antioxidant properties by using a variety of methods and tests, while extracts of the plant are the only currently approved natural antioxidants in the EU (Directive 95/2/EC) and assigned the E number E-392 (European Union Directives 2010/67/EU and 2010/69/EU). According to a new global research study, the worldwide market for rosemary extract is expected to grow at an annual growth rate of roughly 3.7% over the next five years and will reach 260 million US$ in 2024 from 210 million US$ in 2019 [1]. In addition to antioxidant properties, rosemary extracts showed antimicrobial, anti-fungal, bio-plaguicide, anti-inflammatory, anti-carcinogenic activities, as well as therapeutic and prophylactic effects on several physiological disorders [2,3,4,5,6,7]. Most of these activities have been associated with the content in total phenolics or, in particular, carnosic and rosmarinic acid, as well as minor flavonoid constituents.

A body of research focused on the examination of extraction solvents and methods for the recovery of rosemary phenolics. The main parameters that can affect both the yield and the selectivity of the extraction are the extraction solvent, the time, the temperature, the solid-to-liquid ratio, and the granulometry of the raw material. Research efforts focus not only on high-yield extracts rich in bioactive ingredients but also on meeting current energy requirements, such as shorter extraction times, green solvents, and smaller amounts of organic solvents. De AR Oliveira et al. [8] examined mixtures of acetone, methanol, or ethanol with water for the quantitative recovery of rosmarinic acid, carnosol, and carnosic acid and optimized the extraction conditions for the simultaneous extraction of the three compounds with ethanol-water mixtures, through a central composite design. They concluded that, although the optimum yield of each compound varied according to polarity, 70% ethanol in water or 80% acetone in water provided the best additive yield for rosmarinic acid, carnosic acid, and carnosol. Also, ethanol concentrations (30–96%) were studied for the extraction of rosemary leaves through maceration, and 50% ethanol in water showed the highest phenolic yield and antioxidant activity [9]. Ethanol-water mixtures have also been used by other researchers [10,11], as they are considered green solvents. The extraction of carnosic acid in a shaking bath was enhanced with temperature (25–50 °C) and time (30–180 min), while butanone was more effective than ethanol, due to the lower polarity [12].

Among the novel extraction methods, ultrasound assisted extraction (UAE) has been examined by many researchers. Bellumori et al. [13] concluded that the UAE extraction with ethanol or acetone gave high polyphenol yields, and in particular, rosmarinic and carnosic acid, in short times and that UAE extraction can be very favorably compared with the liquid/solid extractions in acetone that are used to prepare commercial rosemary antioxidants. UAE was also found to markedly increase the efficiency of ethanol to extract carnosic acid and to enhance the antioxidant activity of the extract [12,14]. The investigation of the optimal conditions for the extraction of rosmarinic acid, ursolic acid, and oleanolic acid from rosemary leaves by UAE or maceration (90% ethanol, 48 h) led to the conclusion that the highest yield of rosmarinic acid was obtained by UAE with 70% ethanol, of ursolic acid by UAE with 90% ethanol, while maceration gave the highest oleanolic acid yield, and also the TPC yield and antioxidant activity [15].

Accelerated or pressurized solvent extraction, supercritical fluid extraction, or microwave extraction have also been examined [16,17,18,19,20,21], but conventional solid-liquid extraction or UAE still remain effective methods performed at low temperature, cheap, and easy to scale up.

Most of the aromatic plants are mainly commercialized for their essential oil, through steam- or hydro-distillation. Nevertheless, the yield of distillation is rather low, while the solid residue is a potent raw material for the recovery of antioxidants [22,23]. In particular, rosemary yields only 0.3–2.5 g of essential oil per 100 g of the dry plant [11,24,25,26], while a considerable amount of solid residue remains (10–20 × 10^3^ Tn/year) that is currently unexploited [6]. The de-oiled residue results in higher extraction yield and phenolic recovery, probably due to the enhanced penetration of solvent and mass transfer phenomena [27]. However, a few attempts have been published up to now for the utilization of the residue remaining from rosemary essential oil distillation, which currently is discarded and causes environmental concerns. 

The purpose of the present work is initially to exploit the rosemary solid residue, remaining after the recovery of the essential oil, which is usually discarded as waste. In addition, this study aims to define the optimal solid/liquid extraction conditions for antioxidants recovery, in terms of total phenolic content (TPC), as well as individual constituents, through the kinetic study of the extraction parameters. Both ethanol and acetone mixtures in water were used as extraction solvents, and a diffusion-based model was used to predict the yield of TPC and the evolution of individual constituents with time. The effect of temperature on the extraction rate and the phenolics yield obtained by the extraction with a potent acetone-water mixture was examined. Moreover, pre-treatment procedures, namely grinding to various particle sizes, maceration, and pulsed electric fields (PEF) treatments, were examined. Finally, the most promising solvent mixtures were used for UAE, and the results were compared with conventional extraction under stirring. The effect of all the treatments on the recovery of rosemary bioactive compounds was studied.

## 2. Results and Discussion

### 2.1. Effect of Extraction Parameters 

#### 2.1.1. Kinetics of Extraction

**Figure 1:**
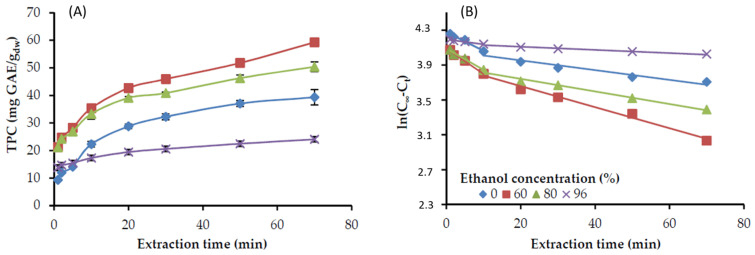
Phenolic compounds extraction from distilled, ground rosemary leaves at different ethanol concentrations in the aqueous solution, 22 °C, and the solid-to-liquid ratio of 1:20 g/mL. (**A**) Experimental values of total phenols recovery (TPC) versus extraction time; (**B**) the factor ln(C_∞_ − C_t_) versus the extraction time, approximation curves based on a diffusion model, according to Equation (1).

**Figure 2:**
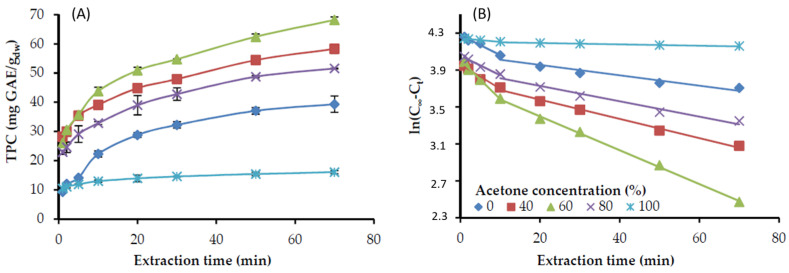
Phenolic compounds extraction from distilled, ground rosemary leaves at different acetone concentration in the aqueous solution, 22 °C, and the solid-to-liquid ratio of 1:20 g/mL. (**A**) Experimental values of total phenols recovery (TPC) versus extraction time; (**B**) the factor ln(C_∞_ − C_t_) versus extraction time, approximation curves based on the diffusion model, according to Equation (1).

The extraction of the rosemary distillation residue was performed at a liquid to solid ratio of 20:1, and constant temperature (22 °C), under continuous stirring. Samples were withdrawn from the reactor at definite time intervals and subjected to analysis for the total phenolic content (TPC) that was expressed as gallic acid equivalents on a dry plant basis (mg GAE/g_dw_). The results of TPC versus time, either with ethanol or acetone aqueous solutions at various concentrations are presented in Figure 1A and Figure 2A, respectively. In all the cases, the concentration of TPC in the solvent solution increased sharply at the beginning of the extraction and leveled off as extraction proceeded to a longer time, approaching equilibrium. 

The extraction of a solute from a plant matrix is generally governed by internal diffusion phenomena. External mass transfer resistance is considered negligible. The concentration of active compounds in the solvent at the interior of the solid particle is equal to the concentration of them in the bulk solvent. The residual concentration of the solute in the solid matrix could be adequately described by Fick’s second law that can be written in a simplified form as [28]:

ln(C_∞_ − C_t_) = ln(C_∞_ − C_0_) − kt (1)

where C_∞_ is the TPC content in the solution obtained at an infinite time (or equilibrium) that was considered equal to 80 mg GAE/g_dw_, according to the maximum TPC values obtained in our experiments. C_t_ is the TPC content in the solution at time t; C_0_ is the TPC content in the solution at time t = 0; k is a rate constant that depends on the diffusion coefficient, and the geometry of the solid particles.

Plotting ln(C_∞_ − C_t_) versus the extraction time resulted in Figure 1B and Figure 2B for the ethanol and acetone solutions, respectively. It is evident that the experimental results are adequately fitted by a two-stage diffusion model, i.e., an initial rapid stage up to approximately 10 min, followed by a slower one until the end of the extraction. The same phenomenon has been observed by other researchers and is usually characterized as a fast extraction step (washing stage) and a slow extraction step (diffusion stage) [28,29]. The rapid stage corresponds to the extraction of the solute from the external sites of the particle, while the relatively slower one corresponds to the diffusion of the solute entrapped in the interior of the plant tissue. The rates of both stages, together with that calculated, through the intercept, C_0_ values are presented in Table 1.

C_0_ indicates a very rapid extraction at the initial contact between fresh solvent and sample [29,30]. It is evident that the initially extracted phenolic components by water, ethanol, or acetone are low, while almost a two-fold increase is observed with ethanol-water mixtures and even more with acetone-water. In terms of kinetics, a suitable extraction solvent can enhance the washing stage and consequently shorten the extraction time. The *k* values indicate that the extraction rate of the fast stage with acetone 60% is the highest, followed by ethanol 60%, while acetone 100% or ethanol 96% presented the lowest rates (Table 1 and Figure 1 and Figure 2). Similar differences were observed among the rates of the slow extraction stage, which were more than two-fold lower that the corresponding fast stage ones. Overall, the two-stage diffusion model could accurately predict the recovery of the TPC from distilled rosemary leaves with either ethanol or acetone solutions.

#### 2.1.2. Effect of Solvent on TPC Recovery 

**Figure 3:**
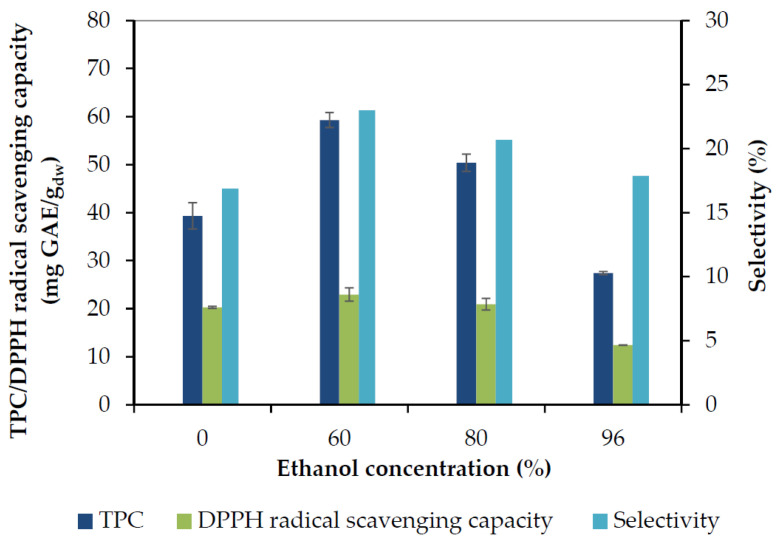
The effect of ethanol concentration on total phenol recovery (TPC), 2,2-diphenyl-1-picrylhydrazyl (DPPH) radical scavenging capacity, and selectivity, obtained after 70 min of extraction.

**Figure 4:**
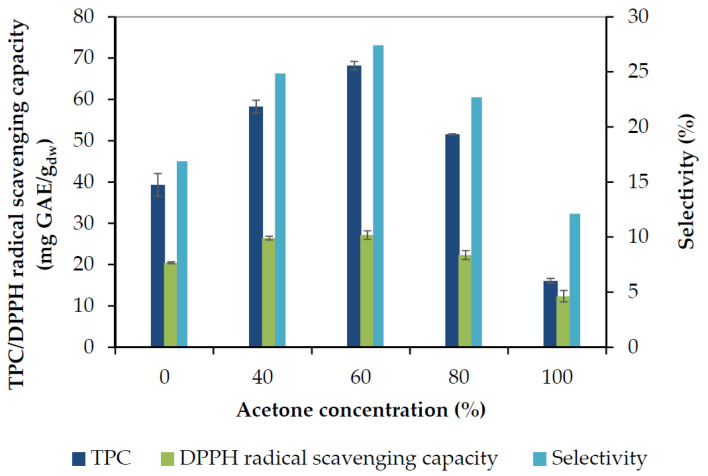
The effect of acetone concentration on total phenol recovery (TPC), DPPH radical scavenging capacity, and selectivity, obtained after 70 min of extraction.

The TPC recovered after 70 min of extraction, together with the obtained DPPH radical scavenging capacity, and the selectivity (TPC/total dried extract yield) of the process performed by the ethanol-water or acetone-water mixtures are presented in Figure 3 and Figure 4, respectively. Rosemary phenolic compounds appear to exhibit enhanced solubility in solvents having intermediate polarity such as aqueous mixtures of alcohols and acetone, in contrast to more polar water or less polar absolute organic solvents.

It is evident that the TPC yield increased with the increase of acetone or ethanol concentration up to a maximum obtained at 60% with either solvent, and decreased afterwards. The antiradical capacity values followed the TPC yield closely, and the same was observed for selectivity. Acetone 60% presented the maximum TPC yield (68 mg GAE/g_dw_), antiradical capacity (27 mg GAE/g_dw_), and selectivity (27%), followed by ethanol 60% and acetone 40%. The pure solvents showed the poorest results. Aqueous organic solvents may extract higher content of phenolics because water may swell the plant material and increase extractability by allowing the organic solvent to penetrate the solid matrix easier. Water is a good solvent for phenolic acids, while aqueous mixtures of organic solvents can also extract the less polar phenolic constituents (as further discussed in Section 2.1.4), which also contribute to the high TPC. The lowest selectivity (12%) was observed with acetone 100% and is due to the low TPC value (16 mg GAE/g_dw_), accompanied by a high amount of non-phenolic compounds extracted with acetone, which is a powerful solvent for several organic compounds.

#### 2.1.3. Effect of Temperature

The effect of temperature (22–60 °C) was studied with acetone 40% that presented a high TPC yield and radical scavenging capacity (Figure 4), while had adequately low acetone content that assured stability under reflux even at 60 °C. The rate of phenolic compounds extraction at the elevated temperatures followed the same two-stage pattern, as at 22 °C, with higher rate constants, k values, as indicated in Table 2. As the temperature increases, the solubility and diffusivity of the solute is enhanced and the transfer of the phenolic components from the plant matrix to the solvent is accomplished in shorter extraction times.

The Arrhenius equation was used to examine the dependence of the rate constant on temperature:

(2) $$k={A\;e}^{\frac{E_{a}}{\mathrm{RT}}}\;\mathrm{or}\;\mathrm{lnk}=\mathrm{lnA}-(\frac{E_{\alpha }}{\mathrm{RT}})$$

where A is a pre-exponential constant, E_α_ is the activation energy (kJ/mol), R is the universal gas constant (8.314 J/(mol K)), and T is the absolute temperature (K).

The Arrhenius plots, i.e., ln(k) versus 1/T, indicated a correlation of k with temperature (R^2^ = 0.76 & R^2^ = 0.99 for the fast and slow extraction stage, respectively (data not shown). The activation energy was calculated through the slope as 22.1 kJ/mol for the fast stage and 8.3 kJ/mol for the slow diffusion stage. These values indicate a much higher dependence of the fast extraction stage on temperature, compared to the slow stage.

Table 2 also presents the final TPC recovery, antiradical capacity, and selectivity at different extraction temperatures. The increase in temperature enhances swelling of the raw material, solubilization, and diffusion of the compounds and, consequently the extraction rate and final yield. Thus, the increase of temperature up to 60 °C resulted in an increase in TPC yield (70.3 mg GAE/g_dw_) and antiradical capacity (31.6 mg GAE/g_dw_), but a decrease in selectivity (20.7). The latter should be attributed to the enhanced extraction of non-phenolic compounds as the temperature increases, or the degradation of some thermo-sensitive active compounds. Also, the increase of temperature facilitates the hydrolysis of ester and ether bonds and, consequently the release of bound phenolics. According to the HPLC analyses, the extraction of rosmarinic acid, which generally presents good temperature stability, was slightly affected by the elevated temperature, while there was no effect on flavonoid extraction, indicating that some degradation may overlap the enhanced extraction of these compounds (Table 2). The number of phenolic diterpenes extracted after 70 min of extraction at the elevated temperature was much higher than at room temperature (25.97 mg GAE/g_dw_ at 60 °C instead of 17.46 mg GAE/g_dw_ at 40 °C and 16.38 mg GAE/g_dw_ at 22 °C). Carnosic acid, although it increases about 35%, as the temperature rises from 22 °C to 40 °C, in agreement with the results of other studies [12], it seems to decrease with further increase in temperature, possibly due to its oxidation to carnosol that rises to very high levels at 60 °C. As carnosol also possesses antioxidant properties, the antiradical capacity of the extracts at 60 °C is also enhanced, despite the fact that carnosic acid is partially degraded. 

#### 2.1.4. Analysis of Phenolic Components and Effect of Solvent on the Phenolic Profile 

HPLC analysis revealed as main components rosmarinic acid, carnosic acid, carnosol, and several minor peaks that corresponded to flavonoids (aglycones and glycosides), according to their UV-Vis spectra. The identification of rosmarinic acid, carnosol, and carnosic acid was performed with the use of internal standards, and the matching of peaks, UV spectra, and retention times. Rosmarinic acid, carnosol, and carnosic acid presented the following retention times in the current study: 38 ± 0.5 min, 53.5 ± 0.5 min, and 58 ± 0.5 min, respectively. In order to identify the flavonoids, the literature was extensively reviewed as far as the elution pattern of flavonoid aglycones and glycosides is concerned [31,32,33,34,35,36,37]. Two zones of eluting flavonoids could be distinguished; the flavonoid glycosides eluted at the first retention time zone, while the flavonoid aglycones at the second. The two zones may overlap at a retention time-frame after the elution of rosmarinic acid.

**Figure 5:**
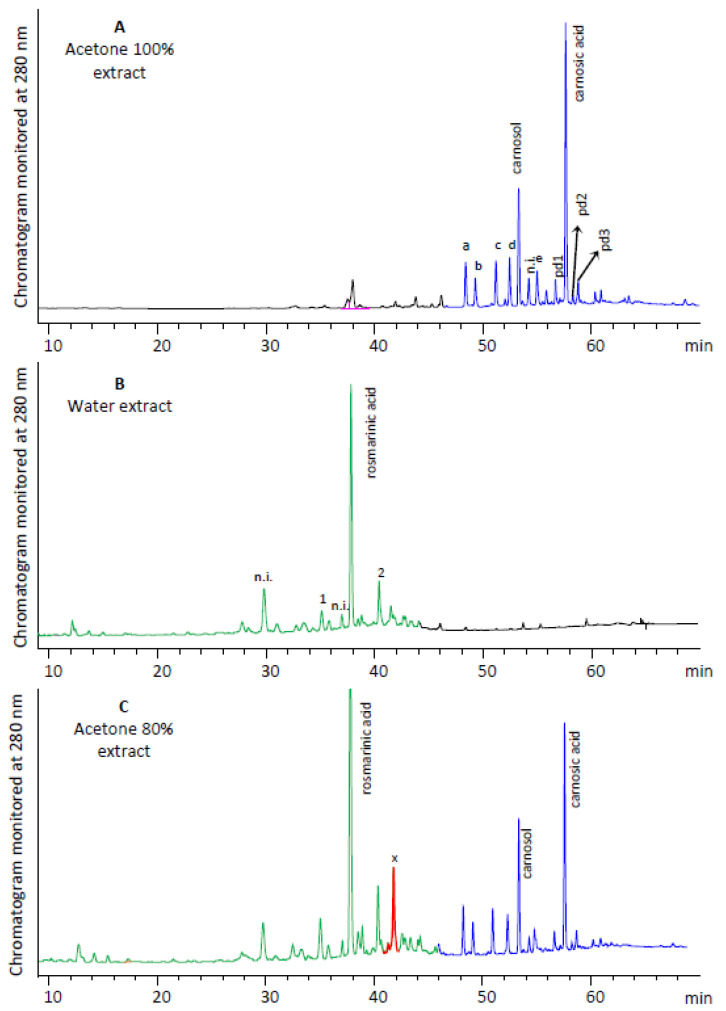
The HPLC-DAD chromatograms of acetone 100% (**A**), water (**B**), and acetone 80% (**C**) extracts, monitored at 280 nm. Blue color indicates the less polar compounds extracted by acetone. Green color indicates the polar compounds extracted by water. Red color corresponds to the compound extracted only by acetone-water mixtures.

The extraction of rosemary with acetone recovered the compounds with the lowest polarity. In the characteristic chromatogram, depicted in Figure 5A, it is apparent that the separated components are eluting at the end of the chromatogram, due to their low polarity. The major component appears to be carnosic acid, followed by carnosol. Three phenolic diterpenes were also detected in traces (pd1, pd2, pd3). Polar compounds such as rosmarinic acid are present in traces. The five peaks “a” to “e” exhibit the characteristic flavone-type UV spectra with the two distinctive absorbance bands: Band A lies in the 310–350 nm range, while band B is found in the 250–290 nm range. The spectra of the detected compounds are presented in Supplementary Figure S1, and their patterns agree with the typical flavone UV-spectra. Since the compounds were recovered by acetone, they are assumed to be flavone aglycones. Acetone is a non-protic solvent that acts as a hydrogen bond acceptor when coming in contact with the phenolics in the plant matrix. Hydrogen bond acceptors have, in general, less ability in forming hydrogen bonds with the compounds to be extracted, except with the phenolic hydrogens, which are more loosely attached to the oxygen [general scheme: Ph-O-H**^…^**O = C(CH_3_)_2_], due to the acid properties of the phenolic molecule. In the case of glycosylated phenolics, apart from steric hindrance effects that might occur, the hydrogen bond between the oxygen of acetone and the alcoholic hydrogens of the saccharide(s) is less stable since the respective hydrogens present far lower acidity (for this reason, acetone is an inappropriate solvent for sugars). 

Between rosemary flavones, it is known that several members contain tri-hydroxy substitution in A-ring, and mono-hydroxy substitution in B-ring, such as scutellarein (4′,5,6,7-hydroxylation), which exhibits UV-spectra similar to compounds **a**, **b**, and **d** [38,39]. However, the retention times of **a**, **b**, and **d** are quite high; therefore, only methylated derivatives of scutellarein could be candidates. 

Hispidulin with 6-mono-methoxy, tri-hydroxy substitution could be a possible structure for peak **a**, since the Band I and II maxima of hispidulin have been reported between 334–38 and 273–275 nm, respectively, and its spectrum pattern is very similar to peak **a** [40,41,42,43]. Peak **b** eluted approximately 1 min after peak **a**, a fact that implies slightly lower polarity than peak **a**. Ladanein is a dimethyl-scutellarein derivative, with methylations at 7- and 4′-hydroxyls, and two maxima at 286 and 335 nm [44], values that are very close to the respective ones of our study (286, 334 nm, Supplementary Figure S1). Furthermore, the patterns of the two UV-spectra are identical, indicating that peak **b** may be identified as ladanein. Compound **d** is clearly less polar than **a** and **b**, eluting more than 3 min after peak **b**. The scutellarein-like UV spectrum presents maxima at 277 and 332 nm. A trimethyl-scutellarein derivative could be a candidate. Martin-Benlloch et al. [44] determined for salvigenin the two maxima at 276 and 332 nm that match our results, as well as the spectrum pattern. Del Pilar Sánchez-Camargo et al. [45] also determined similar values, 276 and 331 nm.

Compound **c** presented apigenin-like spectra and eluted at 50.8 min. Apigenin presents r.t. = 48.6 min, and spectral maxima at 268 and 338 nm, while 4′-methyl-apigenin (acacetin) elutes at 50.5 min (λ_max_ = 268, 334 nm, apigenin-type spectrum). Genkwanin is another mono-methylated apigenin derivative, detected in rosemary [31,33,34,40,46] with average maxima, according to the above references at 268 and 338 nm, equal to those of peak c (Supplementary Figure S1). In agreement with previous analysis of Artemisia extracts that contained the mono-methylated apigenin derivatives, acacetin and genkwanin [47], the higher retention time of peak c (50.8 min) also indicated genkwanin. Compound **e** eluting at 54.8 min, also presents an apigenin-type spectrum with λ_max_ = 268, 332 nm. The characteristic of the compound is that it constitutes the only flavonoid eluting between carnosol and carnosic acid. Previous studies [31,33] have also determined one flavonoid eluting between carnosol and carnosic acid. Both teams have identified the compound as 4′-methoxytectochrysin, and the λ_max_ values they determined are, respectively, 268, 332 nm, and 270, 332 nm, which are almost equal to ours. 4′-Methoxytectochrysin has also been identified by other researchers as the only flavonoid eluting between carnosol and carnosic acid [36,48].

The extraction of rosemary with water recovered the compounds with the highest polarity, and the respective chromatogram is depicted in Figure 5B. The major component is rosmarinic acid, followed by two minor and several components in traces. The minor peaks of water extract with clear UV spectra are presented in Supplementary Figure S1. Compound **1** exhibits a luteolin-type UV spectrum with λ_max_ values determined at 272 and 346 nm. The compound was identified as 6-methoxyluteolin-7-glucoside (nepitrin) due to the λ_max_ values and spectral pattern matching with the literature [41,49,50,51], as well as the expected elution before rosmarinic acid [49,51]. Peak **2** had a flavone-type UV spectrum, with the respective maxima at 268 and 340 nm. The compound was identified as isoscutellarein (4′,5,7,8-tetrahydroxyflavone), which has also been detected by Cuvelier et al. [33] (λ_max_ 268, 340 nm) and Almela et al. [31] (λ_max_ 272, 340 nm) in rosemary extracts, eluting shortly after rosmarinic acid. The elution of isoscutellarein at 40.1 min is in agreement with the above discussion for the elution order of flavones, according to their hydroxylation pattern.

The chromatographic profiles of the extracts recovered by acetone-water mixtures are generally intermediates of pure acetone and pure water extracts. For example, the chromatogram of the 80% acetone extract is depicted in Figure 5C. Apparently the chromatogram almost matches an overlay of a and b, at different ratios of individual peak areas. Only one new compound has emerged, peak **x**, that appears in all the acetone-water extracts. The spectrum of the compound exhibits a pattern detected in specific members of caffeic acid oligomers, such as salvianolic acids. The pattern matches salvianolic acid A, in agreement with the already reported corresponding spectra [52,53]. The ethanol and ethanol-water extractions resulted in similar peak patterns to acetone and acetone-water extracts. Ethanol 96% recovered mainly the phenolic diterpenes and flavone aglycones, a small quantity of rosmarinic acid, and traces of the rest polar components. The ethanol-water extracts presented the pattern of interlaid chromatograms of ethanol 96% and water extracts, such as the acetone-water extracts, with differences in the yields. 

All of the tentatively identified compounds and their content on dry rosemary basis, obtained with the different solvent’s concentrations, after 70 min of extraction, are presented in Table 3. The respective mean retention times (r.t.) of the standard compounds, as reported in literature [31,32,33,34,35,36,37,38,39,40,41,42,43,44,45,46,47,48,49,50,51] and also found in the present work, are also presented.

The highest recovery of carnosic acid was obtained with pure acetone, followed by 80% of either acetone or ethanol. In general, the amount of carnosic acid decreases as we move from acetone to more polar solvents, as it is partially converted to carnosol [8,36], but the total amount of carnosic acid plus carnosol drops down sharply when the water content increases over 40% and 60% in ethanol and acetone solutions, respectively. Rosmarinic acid was the next main component, while salvianolic acid A was found in the extracts of medium polarity, but it was absent or detected in traces in pure solvents. All the flavonoids were detected in much lower concentrations, with isoscutellarein presenting the highest content, among them, in all the extracts.

**Figure 6:**
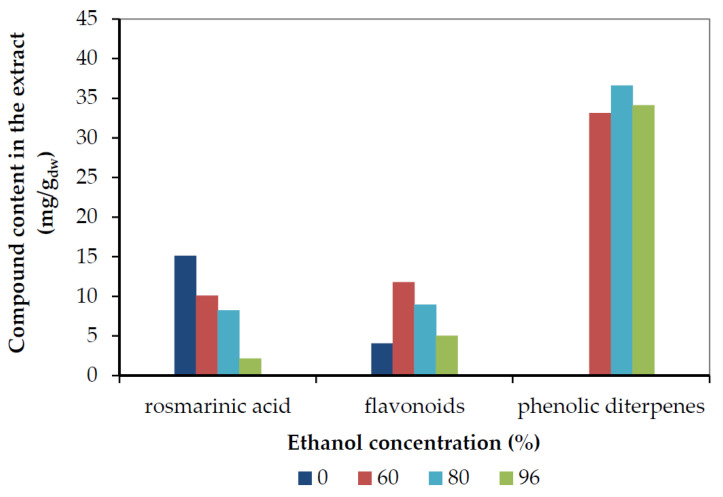
The final recovery of rosmarinic acid, total flavonoids (expressed as mg Que/g_dw_), and phenolic diterpenes (sum of carnosic acid plus carnosol) as a function of ethanol concentration.

**Figure 7:**
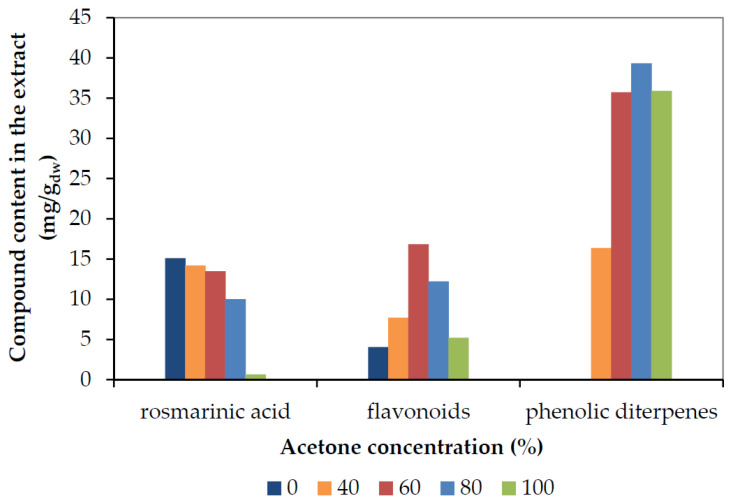
The final recovery of rosmarinic acid, total flavonoids (expressed as mg Que/g_dw_), and phenolic diterpenes (sum of carnosic acid plus carnosol) as a function of the acetone concentration.

Figure 6 and Figure 7 depict the final recovery of rosmarinic acid, total flavonoids (sum of all identified flavonoid aglycones and glycosides), and phenolic diterpenes (sum of carnosic acid, carnosol, and minor diterpenes) as a function of ethanol and acetone concentration, respectively.

As can be seen, the maximum rosmarinic acid was recovered with pure water, while its yield decreased with the increase in the concentration of either solvent. Pure acetone or ethanol extracted very low amounts of rosmarinic acid after 70 min. Rosmarinic acid, as a polar compound is easily solubilized in water and removed from the plant matrix, while the organic solvent concentration increases, its solubility decreases, and, thereof, the recovery in condensed solvents could be enhanced by prolonging the extraction duration or increasing the extraction temperature [10]. On the contrary, phenolic diterpenes are not soluble in water and their highest yields were obtained at 80% concentration with either solvent. These results are in agreement with de AR Oliveira et al. [8], who reported that 80% acetone quantitatively recovered the main antioxidants of rosemary (namely rosmarinic acid, carnosic acid, and carnosol), while 59% and 70% ethanol also showed very close recovery. It should be noted, additionally, that they observed quantitative recovery of rosmarinic acid with water, too. The maximum total flavonoids recovery was obtained with a concentration of 60% by either solvent. This result is due to the presence of non-polar flavonoid aglycones, as well as medium polarity flavonoid glucosides, as shown in Table 3. The recovery of the individual flavonoids as a function of ethanol and acetone concentration is depicted in Supplementary Figures S2a,b (Supplementary Files). Isoscutellarein and nepitrin presented clear maxima at 60% of either solvent, as they are compounds of medium polarity, while they were not detected in pure organic solvents. All the rest compounds had a considerably lower content in the extracts and presented smaller differences with solvent concentration, except water that did not recover any of them.

The experimental data of rosmarinic acid, phenolic diterpenes (carnosic acid plus carnosol), and total flavonoids (sum of all identified flavonoid compounds) versus the extraction time were further analyzed by the two-stage diffusion model, as described in 2.1.1. A good correlation was obtained in all cases with the *k* values, depending on the compound and solvent (Supplementary Table S1). The highest extraction rates (*k* values) of rosmarinic acid were obtained with water, and decreased as the ethanol or acetone content in the solvent increased to minimum, when ethanol 96% or acetone 100% were used as solvents. The higher rates of the total flavonoids extraction were obtained with ethanolic solution 60% or acetonic solution 40–80%. The phenolic diterpenes showed close extraction rates with any concentration of the organic solvents, though the highest values were obtained with 80% acetone. These results are in agreement with the polarity and solubility of the compounds—Rosmarinic acid, being the most polar and water-soluble compound, is readily extracted with water; the detected flavonoids show medium polarity; thus their extraction is accelerated by medium polarity solvents; the phenolic diterpenes are non-polar compounds, with a high acetone-solubility but a low percentage of water accelerates their extraction due to swelling of the raw material. 

### 2.2. Effect of Pre-Treatment

#### 2.2.1. Grinding/Milling

**Figure 8:**
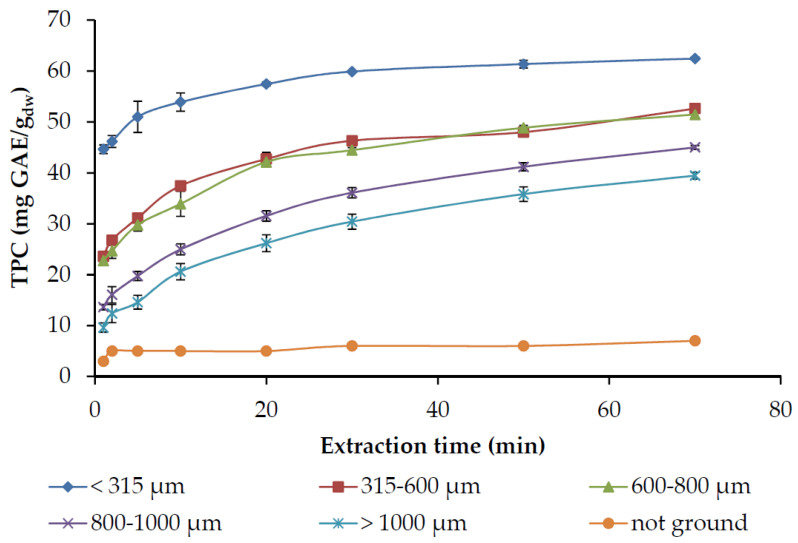
The influence of grinding to different particle sizes on the total phenols recovery (TPC) by 80% acetone in water solution, at 22 °C, and solid-to-liquid ratio of 1:20 g/mL.

The effect of grinding was examined by using rosemary needles remaining after the essential oil distillation, either not ground or ground to several particle sizes. The extractions were performed with acetone 80% that proved the most effective solvent, and the results are presented in Figure 8 and Table 4. It is evident that the rate of phenolic compounds extraction from the non-ground material was extremely low. As the extraction is governed by inner mass transfer phenomena, grinding to a smaller particle size results in smaller diffusion distance and larger diffusion area, and, thereof, an increased extraction rate and reduced extraction time required. More specifically, as can be seen from Figure 8, the extraction of smaller particles approaches the maximum equilibrium concentration, almost after 30 min, earlier than particles of larger size. The rate constant of the fast stage also increased substantially as the particle size of the ground material decreased (Table 4). Similarly, the number of phenolic compounds that are readily extractable (C_0_) increased as the particle size decreased, a fact that can be explained by the release through the grinding of the solutes close to the surface. 

The rate of the slow stage was not affected by the different particle size obtained through grinding. The TPC obtained after 70 min of extraction, and consequently, the antiradical capacity were increased as the particle size decreased due to the higher overall rate. Rodríguez-Rojo et al. [54] also observed increased TPC recovery after grinding and commented that the milling process reduced inner mass transfer limitations. The selectivity of the process does not seem to be related to particle size, although a higher value was obtained at the smallest particle size, probably because the extraction of phenolics is considerably enhanced in the smallest particles (Figure 8). The HPLC analysis of the extracts indicated that the consistency in individual components was not significantly different among the fractions with different particle sizes (data not shown). 

#### 2.2.2. Maceration and PEF

**Figure 9:**
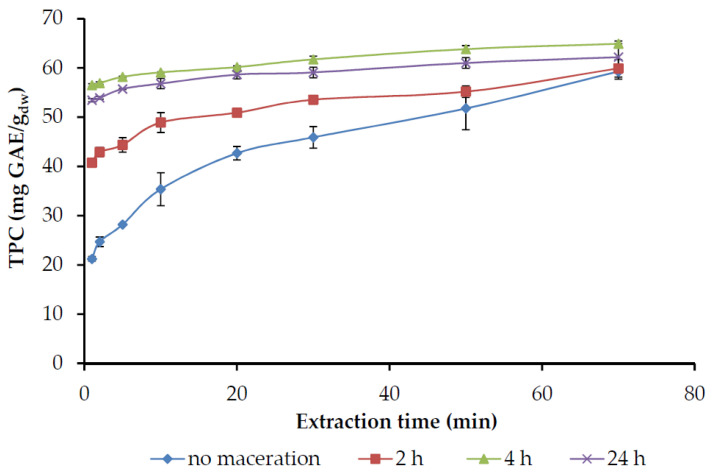
The influence of maceration time of ground rosemary leaves in cold water, on total phenols recovery (TPC), through extraction by ethanol 60%, at a solid-to-liquid ratio of 1:20 g/mL.

Figure 9 shows the effect of maceration in water for 2, 4, or 24 h, at room temperature, as well as maceration combined with PEF on the extraction of phenolic compounds by 60% ethanol. 

Maceration causes swelling of the material and dissolution of the phenolic compounds; therefore, results in higher C_0_ (Table 4). These rapidly dissolving components giving rise to the intercept are actually solutes in the leaf close to the surface, rather than components adhering to the outside of the solid [55]. Two h maceration treatment resulted in doubling C_0_ value, while the maximum was obtained after 4 h. The phenolic recovery, antiradical activity or selectivity recovery were also maximized with 4 h of maceration while extending the duration of the treatment did not cause any further improvement. This can be attributed to the fact that after a few hours, the increase in concentration in the aqueous phase has reached a level where the driving force of mass transfer becomes zero. The extraction rate of the macerated material was lower (Table 4), since the—to be extracted—phenolic components that remain in the plant cells have been reduced. The results indicate that the quantitative recovery of phenolic compounds could be achieved in shorter extraction time if the raw material is subjected to maceration for 4 h prior to extraction. The same yields were achieved after 10–20 min of extraction, instead of 70 min, resulting in a less energy-intensive process. 

Although there are several studies concerning the enhancement of bioactive compound extraction, by the use of PEF, in our case, PEF processing did not provide any effect on the extraction of the macerated material (Table 4). The focus of applications of PEF is to make cell membranes permeable to improve the transfer of components from the inside of the cells. Significant enhancement of the phenolic extractability in the solid-liquid extraction of spearmint was observed when working at a PEF intensity corresponding to 99 pulses of 3 kV/cm with a specific energy input of 4102 ± 239 J/kg [56]. The efficiency of PEF processing of tea leaves is related to both the electric field and the relaxation time after a series of pulses, i.e., when the electric field strength is 1.1 kV/cm, it takes 3 s to achieve the desired extraction efficiency [57]. Moubarik et al. [58] noted that a further increase in PEF intensity from 0.35 kV/cm to 0.43 kV/cm did not increase the extraction kinetics of fennel. Consequently, there is a specific value of PEF intensity for each material so as to achieve the maximum permeability of cellular membranes. As there are no reports for rosemary treatment with PEF, this needs further investigation. 

### 2.3. Ultrasound-Assisted Extraction

UAE was performed by using the solutions that presented the highest yields, i.e., acetone 80%, acetone 60%, and ethanol 60%. The increase of phenolic compounds in the solution followed the same pattern, as in the agitated reactor but with a considerably higher rate. The obtained TPC yield and the antiradical activity were increased, and the results are presented in Table 5. In particular, the highest improvement by UAE was obtained with ethanol 60% as solvent, in agreement with the results of other studies [12,18], who observed that the efficiency of ethanol was substantially increased with the use of UAE. This fact can be explained by the lower penetration and solubilization ability of ethanol, compared to acetone. Thus, the effect of UAE is more pronounced for ethanol solutions, compared to acetone ones. 

The mechanism for ultrasonic enhancement can be ascribed to an intensification of mass transfer arising from the collapse of cavitation bubbles near the cell walls. Ultrasound can break down the cell walls, and as a result, better contact between solvent and plant material will take place. Further, when the cavitation bubbles collapse, an ultrasonic jet is produced and will act as a solvent micro-pump that can force a solvent into the cell to dissolve the components [12]. 

Similar recovery of total and individual phenolic compounds was achieved after 10 min of UAE, instead of 70 min of conventional agitated extraction. Several studies observed that the UAE resulted in a meaningful shortening of processing time at about 10–12 min [18,59,60]. In terms of individual compounds, the use of UAE had a small effect on the phenolic profile of the extracts, depending on the solvent. The most significant increase in individual phenolic concentration was observed with ethanol 60% (Table 5), while the same solvent, under UAE, also enhanced the rate of extraction of rosmarinic acid, flavonoids, and phenolic diterpenes (Supplementary Table S1). The rosmarinic acid content was remarkably increased (1.6 times) when ethanol 60% was used in UAE, while a smaller increase was also observed with the acetone-water mixtures. Carnosic acid extraction showed a similar increase of about 20% with all the three solvent mixtures tested. The acetone-in-water mixtures; however, appear to depress flavonoid recovery. Dent et al. [60] confirmed that the difference in polarities of the extracting solvents influences the solubility of the chemical constituents, finding that ethanol 30% showed a greater enhancement in the mass fraction of flavonoids compared with acetone 30%. It is characteristic that selectivity was also slightly decreased in the case of 60% acetone in UAE. 

## 3. Materials and Methods

### 3.1. Solvents and Reagents

Ethyl alcohol and acetone, used for the extraction process, were obtained from Sigma Aldrich (Steinheim, Germany). The materials used for the analysis of the extracts were Folin Ciocalteu phenol reagent (2N) obtained from Merck (Darmstadt, Germany), sodium sufhate anhydrous (>99%) from Mallinckrodt (St. Louis, MS, USA), 2,2-diphenyl-1-picryl hydrazyl (DPPH) from Sigma-Aldrich (Steinheim, Germany) and gallic acid (98% *w/w*) from Acrōs Organics (Fair Lawn, NJ, USA). The standard compounds used in the study were quercetin dihydrate and rosmarinic acid, products of Sigma-Aldrich (Steimheim, Germany). Carnosic acid was obtained from Dayang Chemicals Co (Hangzhou, China), and carnosol from Extrasynthese (Lyon, France). Water, acetonitrile, methanol, and trifluoroacetic acid for LC-MS analyses were obtained from Fisher Chemical (Leicestershire, UK). 

### 3.2. Plant Material

The rosemary used for the experiments was a commercial organic plant (Farm Bioma, Aridea-Thessaloniki, Greece), cultivated and harvested in Kilkis, Northern Greece, by the company Organic Islands (Naxos, Greece). The batch number of the provided material was RMA003170610, while a voucher sample has been kept at the Laboratory of Food Chemistry and Technology. The plant was subjected to water-steam distillation for 6 h, so as to remove the essential oil, as described by Tsimogiannis et al. [61]. The wet herbal residue was dried in a ventilated oven (Function Line UT20, Heraeus Instruments GmbH, Hanau, Germany) at 35 °C for 24 h, and ground with a high-speed household blender.

### 3.3. Pretreatment Procedures

#### 3.3.1. Grinding

To examine the effect of grinding, a batch of the ground material was fractionated by using a vibrating sequence of sieves of different aperture sizes (BA200N, Cisa, Barcelona, Spain). Sieves of aperture size 315 μm, 600 μm, 800 μm, 1000 μm were used and the obtained fractions (i.e., particle size <315, 315–600, 600–800, 800–1000, and >1000 μm) were used for extraction. Additionally, a batch of distilled, not ground material was used.

#### 3.3.2. Immersion/Maceration

The ground material (5 g) was immersed in distilled water (20 mL), and kept at room temperature for 2, 4, or 24 h. Consequently, the mixture was transferred to the extraction vessel and subjected to extraction.

#### 3.3.3. Pulsed Electric Fields (PEF) 

The ground plant material (5 g) was first immersed in distilled water for 4 h, at a solid-to-liquid ratio of 1:4, so as to become fully wet. It was then subjected to PEF treatment in a laboratory apparatus for food processing (Elcrack-5 kW, DIL, Quakenbrück, Germany). The sample was placed in a treatment chamber between two stainless steel electrodes with a gap width of 4 cm, and a certain amount of water was added until the chamber was filled (approximately 60 mL). The field strength of 5.2 kV/cm was applied, with the simultaneous production of 1000 pulses of 15 μsec duration each. The received suspension was then transferred to the extraction vessel, the appropriate amount of solvent was added in order to achieve a final concentration of 60% ethanol and the mixture was subjected to extraction.

### 3.4. Extraction Procedure

#### 3.4.1. Conventional Solid/Liquid Extraction under Stirring

The extraction of the phenolic compounds was performed into a spherical extraction vessel equipped with a multiple-neck lid bearing a vertical water cooler. The vessel was placed in a temperature-controlled (±1.0 °C) water bath, and continuously agitated with a propeller-type stirrer, operating at 200 rpm (R7R1, Heidolph, Schwabach, Germany). The material (5 g) was placed in the vessel, and the appropriate amount of the solvent, preconditioned at the desired temperature (22, 40, or 60 °C), was added, so as the final solid-to-liquid ratio to be 1:20 g/mL, which proved efficient for the recovery of phenolic antioxidants [62]. Samples of 1 mL were removed with a pipette at definite time intervals, filtered immediately upon receipt, and processed for analysis. At the end of the extraction, the extract was filtered, its volume was measured and the solid residue was determined, by drying duplicate 5 mL-samples in an oven at 103 °C. All experiments were run in duplicate, and the presented results are mean values.

#### 3.4.2. Ultrasound Assisted Extraction (UAE)

For the UAE, the reaction vessel used in 3.4.1, without the stirrer, was immersed in an ultrasonic bath (Elmasonic S, Elma, Schmidbauer, Germany), equipped with an ultrasonic frequency of 37 kHz. The temperature of the bath was maintained at 22 °C. 

### 3.5. Determination of Total Phenol Content 

The total phenolic content (TPC) of the extracts was determined by the Folin-Ciocalteu reagent using the method of Singleton et al. [63]. The absorbance of all samples was measured at 765 nm using a T90+ UV-Vis Spectrometer (T90+, PG Instruments, Leicestershire, England). Duplicate measurements of each extract were performed and averaged. The results are expressed as gallic acid equivalents on the dry plant basis (mg GAE/gdw), through the construction of a calibration curve obtained with authentic reference compound (gallic acid).

### 3.6. DPPH Free Radical Scavenging Assay

The antiradical activity of the samples collected from the different extraction procedures was determined by the DPPH radical assay. A UV-Vis instrument (T90+, UV-Vis Spectrometer, PG Instruments, Leicestershire, England) was used to monitor the reaction of DPPH radical with the sample. Samples (0.1 mL) of extract solution, appropriately diluted in methanol, were added to 3.9 mL of 6 10^−5^ M DPPH radical solution in methanol, and the absorbance at 515 nm was recorded after 30 min, according to the methodology reported by Brand-Williams et al. [64]. Duplicate measurements of each extract were performed and averaged. The results are expressed as gallic acid equivalents on the dry plant basis (mg GAE/g_dw_), through the construction of a calibration curve obtained with authentic reference compound (gallic acid).

### 3.7. Selectivity

The solid residue of the extracts was determined through drying in a laboratory oven at 103 °C, as described in 3.4.1. Duplicate measurements of each extract were performed and averaged. The selectivity (%) of the extraction is defined as the percentage of the total phenolic content (TPC) on the solid residue of the extract.

### 3.8. HPLC-DAD Analyses

The high-performance liquid chromatography with a diode-array detector (HPLC-DAD) method proposed by Merken and Beecher [65], and modified by Tsimogiannis et al. [66] was used in order to detect the main phenolic compounds of the extracts. The HPLC apparatus consisted of an HP 1100 gradient pump and a diode array detector (Hewlett Packard, Waldbronn, Germany). A ZORBAX Eclipse XDB-C18 column (5 µm, 250 × 4.6 mm, Agilent, Santa Clara, CA, USA) was used under thermostated conditions at 30 °C. The samples were injected after filtration (0.45 μm, PVDF syringe filters, Teknokroma, Barcelona, Spain), and the flow rate was 1 mL/min. The solvent system consisted of water (**A**), methanol (**B**), and acetonitrile (**C**), each containing 0.2% trifluoroacetic acid. The initial composition of the mobile phase was 90% **A**, 6% **B**, and 4% **C**. With linear gradients, the composition changed to 85% **A**, 9% **B**, and 6% **C** within 5 min, 71% **A**, 17.4% **B**, and 11.6% **C** within 30 min, and 0% **A**, 85% **B**, and 15% **C** within 60 min. The injection volume was 20 µL, while the elution of compounds was monitored at 280 and 360 nm. System control, data acquisition, and data processing were performed using the Varian Workstation (Varian Inc., Palo Alto, CA, USA). The identification of compounds was performed according to the UV-Vis spectra of the peaks, retention times, use of internal standards, and comparison with literature data. Rosmarinic acid was quantified according to the calibration curve obtained with the respective authentic reference compound (rosmarinic acid), while flavone aglycones and glycosides were quantified as quercetin equivalents according to the calibration curve obtained with the respective authentic reference compound (quercetin). Both rosmarinic and quercetin calibration curves were obtained in the range of 20–200 mg/L analyzing four concentrations (20, 50, 100, 200 mg/L) from duplicate samples. The detection of compounds was performed at the previously mentioned wavelengths and the produced linear functions presented R^2^ > 0.996. 

Despite the fact that the phenolic diterpenes were very well separated by the above method, the known instability of carnosic acid in organic solvents and the high retention of the phenolic diterpenes in the column [65] led us not to use the specific method for the quantifications of phenolic diterpenes. The phenolic diterpenes were quantified by the method proposed by Okamura et al. [67] with a ZORBAX Eclipse XDB-C18 column (5 µm, 250 × 4.6 mm, Agilent, Santa Clara, CA, USA). Phosphoric acid (50 mg/L) was added to the extract samples to retain carnosic acid [68]. The analysis was performed under isocratic conditions with 0.1% phosphoric acid and 60% acetonitrile as the mobile phase at a flow rate of 1.0 mL/min and detection at 230 nm. The quantification of individual phenolic diterpenes was based on the respective calibration curve obtained with authentic reference compound (carnosic acid). The stock solution of carnosic acid was prepared in duplicate at a concentration of 200 mg/L, using acidified 2-propanol with phosphoric acid (0.1%). The stock solutions were diluted to 100, 50, and 20 mg/L, and all samples were analyzed according to the method described above. The linear function of peak areas at 230 nm with the concentration of carnosic acid presented R^2^ = 0.9999. 

No specific concentration of extract was used for the HPLC-DAD analyses. Each extract was diluted appropriately, so as the peaks to be quantified to meet the ranges of the respective calibration curves of the respective authentic reference compounds.

## 4. Conclusions

Consumers’ keen interest in bioactive ingredients from natural raw materials, as well as their growing concern about the environmental impact of waste from many physicochemical processes, is constantly leading studies to isolate the above ingredients from natural by-products. The residual material from rosemary after the removal of its essential oil proves to be rich in phenolic ingredients with strong overall antioxidant capacity. Extracts with rich phenolic content were recovered by using environmentally friendly solvents, such as aqueous mixtures of ethanol and acetone, and enhancing the extraction process by pretreatment methods such as grinding and maceration. 60% ethanol or acetone in water solution resulted in the highest extraction rates, TPC recovery, and antioxidant capacity while increasing temperature from 22 °C to 60 °C resulted in increased extraction efficiency and antioxidant activity. UAE extraction was found to enhance the TPC yield and the antioxidant capacity of the extracts, especially when ethanol 60% was used as a solvent. Additionally, this study focused on the extraction kinetics of both total phenols and individual compounds recovered from rosemary. It was evident that extraction proceeds at a high rate initially and, at approximately 10 min, slows down to level off before reaching equilibrium. The HPLC analysis indicated mainly the presence of rosmarinic acid, flavonoids and phenolic diterpenes (carnosic acid, carnosol). Water extract showed the highest content of rosmarinic acid, while the solution with acetone content of 60% exhibited the highest flavonoid recovery and the one with acetone content of 80% the highest phenolic diterpenes yield. Based on the results of the present work, residues of rosemary essential oil distilleries could be exploited for the recovery of phenolic antioxidants. Conventional solid-liquid extraction with appropriate pretreatments, or novel extraction methods, such as UAE, can be used. Further study should focus on meeting the modern requirements for limiting waste and reducing energy, which often acts as a deterrent to the application of the above methods on an industrial scale.

## Figures and Tables

**Table 1:** The initial concentration of phenolics in the solvent (C_0_) and the rate constant (k) of each extraction step, performed by different aqueous solutions of ethanol or acetone, at 22 °C, solid-to-liquid ratio 1:20, and non-specific particle size, as calculated by fitting Equation (1). The correlation coefficients (R^2^) are also presented.

	C_0_ (mg GAE/g_dw_)	k (min^−1^) (Fast Stage)	R^2^	k (min^−1^) (Slow Stage)	R^2^
**Ethanol Concentration (%)**					
**0**	7.96 ± 1.02	0.022 ± 0.002	0.97	0.006 ± 0.001	0.93
**60**	20.30 ± 0.80	0.029 ± 0.003	0.99	0.012 ± 0.002	0.99
**80**	20.43 ± 0.76	0.024 ± 0.002	0.98	0.007 ± 0.001	0.98
**96**	13.65 ± 0.22	0.006 ± 0.001	0.98	0.002 ± 0.001	0.97
**Acetone Concentration (%)**					
**0**	7.96 ± 1.02	0.022 ± 0.002	0.97	0.006 ± 0.001	0.93
**40**	27.48 ± 1.14	0.026 ± 0.004	0.96	0.010 ± 0.001	0.99
**60**	24.95 ± 1.02	0.042 ± 0.003	0.99	0.018 ± 0.002	1.00
**80**	22.23 ± 0.83	0.021 ± 0.003	0.97	0.008 ± 0.001	0.97
**100**	10.39 ± 0.18	0.004 ± 0.001	0.97	0.002 ± 0.001	0.96

**Table 2:** The effect of temperature on the initial concentration of phenolics in the solvent (C_0_), the rate constant (k) of each extraction step, the total phenolic content (TPC), antiradical capacity, selectivity, rosmarinic acid, total flavonoids, carnosic acid, and carnosol, recovered after 70 min of extraction performed with acetone 40%, at a solid-to-liquid ratio 1:20 and non-specific particle size. The correlation coefficients (R^2^) of fitting Equation (1) to experimental data are also presented.

	Temperature (°C)		
	22	40	60
C_0_ (mg GAE/g_dw_)	27.48 ± 1.14	28.64 ± 0.65	28.60 ± 1.92
k (min^−1^) (fast stage)	0.026 ± 0.004	0.027 ± 0.002	0.074 ± 0.006
R^2^ (fast stage)	0.96	0.99	0.99
k (min^−1^) (slow stage)	0.010 ± 0.001	0.013 ± 0.001	0.015 ± 0.002
R^2^ (slow stage)	0.99	0.98	0.95
Total Phenol Content (mg GAE/g_dw_)	58.3 ± 1.5	61.7 ± 0.4	70.3 ± 1.0
DPPH radical scavenging capacity (mg GAE/g_dw_)	26.7 ± 0.5	28.5 ± 0.6	31.6 ± 0.7
Selectivity (%)	24.9	22.9	20.7
Rosmarinic acid (mg/g_dw_)	11.12 ± 0.6	12.76 ± 0.8	12.97 ± 0.5
Total flavonoids (mg Que */g_dw_)	7.72 ± 0.4	7.70 ± 0.3	7.36 ± 0.8
Carnosic acid (mg/g_dw_)	10.79 ± 0.5	14.49 ± 0.3	13.60 ± 0.7
Carnosol (mg CA */g_dw_)	5.57 ± 0.1	2.96 ± 0.6	12.35 ± 0.5

* Que: quercetin equivalents, CA: carnosic acid equivalents.

**Table 3:** Tentative identification of the phenolic components of the extracts obtained by different ethanol or acetone concentration, after 70 min of extraction, at 22 °C, solid-to-liquid ratio 1:20, and non-specific particle size.

Coding	Identified Compounds	r.t.	Recovery of Compounds (mg/g_dw_) in								Reference
		(min)	Acetone in Water Extracts				Water Extract	Ethanol in Water Extracts			For UV and r.t. Data
			100%	80%	60%	40%		60%	80%	96%	
1	nepitrin ^1,a^	35.2	0	2.20	3.01	2.66	2.05	3.01	2.25	0	[41,49,50,51]
	rosmarinic acid ^b^	38.0	0.66	10.02	13.49	14.20	15.13	10.11	8.24	2.16	-
2	isoscutellarein ^1,a^	40.1	0	2.58	5.97	4.43	3.97	4.90	3.23	0	[31,33]
x	salvianolic acid A ^2,a^	41.8	0	3.51	6.23	5.11	tr	3.79	3.25	0	[52,53]
a	hispidulin ^1,a^	48.0	1.24	1.69	1.58	1.17	0	1.62	1.54	1.23	[40,41,42,43]
b	ladanein ^1,a^	49.0	0.93	1.14	1.20	1.45	0	1.04	1.11	0.96	[44]
c	genkwanin ^1,a^	50.8	1.30	1.56	1.48	1.19	0	1.33	1.41	1.34	[31,33,34,40,46,47]
d	salvigenin ^1,a^	52.2	1.38	1.47	1.37	1.34	0	1.31	1.35	1.30	[44,45]
	carnosol ^3,b^	53.5	7.55	13.83	14.69	5.57	0	7.38	10.48	10.19	-
e	4′-methoxytectochrysin ^1,a^	54.8	1.23	0.99	1.37	0.82	0	1.06	1.16	1.33	[31,33,36,48]
	carnosic acid ^b^	58.0	26.54	23.84	19.37	10.79	0	10.18	24.43	22.28	-

^1^ expressed as quercetin equivalents; ^2^ expressed as rosmarinic acid equivalents; ^3^ expressed as carnosic acid equivalents; ^a^ Assginment is based on comparison with UV- and retention time data from literature and hence tentative; ^b^ Identified by direct comparison of retention time and UV spectrum with authentic reference samples.

**Table 4:** The effect of grinding to different particle size, maceration at different duration, or maceration combined with Pulsed Electric Field (PEF) on the extraction model parameters, total phenolic content (TPC), antiradical capacity, and selectivity.

	C_0_ (mg GAE/g_dw_)	k (Fast Stage) (min^−1^)	R^2^	k (Slow Stage) (min^−1^)	R^2^	TPC (mg GAE/g_dw_)	Antiradical Capacity (mg GAE/g_dw_)	Selectivity (%)
Particle Size (μm) *								
D < 315	44.10 ± 1.09	0.034 ± 0.005	0.95	0.006 ± 0.001	0.88	62.5 ± 0.3	33.6 ± 1.1	28.3
315 < D < 600	22.76 ± 0.71	0.030 ± 0.002	0.99	0.007 ± 0.001	0.94	52.6 ± 0.5	31.9 ± 0.9	18.9
600 < D < 800	22.05 ± 0.95	0.024 ± 0.003	0.97	0.007 ± 0.001	0.92	51.5 ± 0.6	29.2 ± 0.2	18.2
800 < D < 1000	12.95 ± 0.54	0.020 ± 0.001	0.99	0.007 ± 0.001	0.97	45.0 ± 0.4	20.6 ± 0.7	19.8
1000 < D	8.96 ± 0.71	0.018 ± 0.002	0.98	0.006 ± 0.001	0.97	39.5 ± 0.6	19.1 ± 0.0	18.4
Not ground	3.87 ± 0.82	0.002 ± 0.001	0.33	0.001 ± 0.001	0.89	7.0 ± 0.2	23.1 ± 1.1	22.7
Maceration (h)/PEF **								
0	20.30 ± 0.80	0.029 ± 0.003	0.99	0.012 ± 0.002	0.99	59.3 ± 1.5	23.9 ± 1.4	23.0
2	40.26 ± 0.62	0.024 ± 0.003	0.98	0.007 ± 0.002	0.97	59.9 ± 1.5	31.2 ± 0.8	25.4
4	56.37 ± 0.25	0.013 ± 0.002	0.96	0.006 ± 0.001	0.98	64.9 ± 1.0	34.8 ± 1.1	31.1
24	53.25 ± 0.38	0.015 ± 0.002	0.95	0.004 ± 0.001	0.98	62.2 ± 0.5	32.3 ± 0.7	26.0
4 + PEF	54.67 ± 0.24	0.014 ± 0.002	0.97	0.005 ± 0.001	0.88	64.0 ± 0.3	34.9 ± 1.0	26.7

* Extraction performed by acetone 80%, at 22 °C and solid-to-liquid ratio 1:20 g/mL; ** Extraction performed by ethanol 60%, at 22 °C, solid-to-liquid ratio 1:20 g/mL, and non-specific particle size.

**Table 5:** The effect of Ultrasound Assisted Extraction (UAE) on the extraction model parameters, total phenolic content (TPC), antiradical capacity, selectivity, rosmarinic acid, total flavonoids, and total phenolic diterpenes (sum of carnosic acid plus carnosol) recovered after 70 min of extraction by using different solvents.

Measured Parameter	60% Ethanol		60% Acetone		80% Acetone	
	Agitation	UAE	Agitation	UAE	Agitation	UAE
k_fast_ (min^−1^)	0.029 ± 0.003	0.142 ± 0.011	0.042 ± 0.003	0.077 ± 0.008	0.021 ± 0.003	0.059 ± 0.008
k_slow_ (min^−1^)	0.012 ± 0.002	0.043 ± 0.003	0.018 ± 0.002	0.027 ± 0.008	0.008 ± 0.001	0.020 ± 0.005
TPC (mg GAE/g_dw_)	59.3 ± 1.5	77.5 ± 1.2	68.2 ± 1.0	73.4 ± 0.7	51.5 ± 0.1	67.5 ± 0.4
Antiradical capacity (mg GAE/g_dw_)	23.9 ± 1.4	37.8 ± 1.1	27.9 ± 1.0	34.4 ± 0.9	23.1± 1.1	28.7 ± 1.0
Selectivity %	23.0	29.9	27.4	26.8	22.7	28.1
Rosmarinic acid (mg/g_dw_)	10.1 ± 0.6	16.0 ± 0.8	13.5 ± 0.5	16.6 ± 0.4	10.0 ± 0.8	14.1 ± 0.7
Total flavonoids (mg Que */g_dw_)	11.8 ± 0.4	16.1 ± 0.3	16.8 ± 0.7	14.9 ± 0.7	12.2 ± 0.4	11.6 ± 0.6
Carnosic acid (mg/g_dw_)	24.2 ± 0.6	29.1 ± 0.9	19.4 ± 0.4	23.0 ± 0.8	23.8 ± 0.4	27.5 ± 0.6
Carnosol (mg CA */g_dw_)	10.5 ± 0.5	16.1 ± 0.7	14.7 ± 0.3	10.9 ± 0.6	13.8 ± 1.0	15.5 ± 0.5
Total phenolic diterpenes (mg/g_dw_)	33.1 ± 0.8	47.4 ± 1.1	35.7 ± 1.0	38.8 ± 0.9	39.3 ± 1.3	45.0 ± 1.1

* Que: quercetin equivalents, CA: carnosic acid equivalents.

## Supplementary Materials

The following are available online. Table S1: The effect of the ethanol or acetone concentration (%) and of Ultrasound Assisted Extraction (UAE) on the initial concentration (C0) and the rate constant (*k*) of each extraction step of rosmarinic acid, total flavonoids and total phenolic diterpenes (sum of carnosic acid plus carnosol), as calculated by fitting Equation(1). The correlation coefficients (R2) are also presented, Figure S1: The UV-Vis spectra of the minor peaks detected in the chromatograms of the water, acetone 100%, and water-acetone extracts, Figure S2: The recovery of the individual identified flavones with the increase of ethanol (**a**) and acetone (**b**) in extraction solvent.
