# Supplementary material for: Extraction Kinetics of Phenolic Antioxidants from the Hydro Distillation Residues of Rosemary and Effect of Pretreatment and Extraction Parameters
Source: Molecules. 2020 Oct 2;25(19):4520. doi: 10.3390/molecules25194520 (PMC7582955; doi:10.3390/molecules25194520)
Supplement: Supplementary file 1 [file molecules-25-04520-s001.pdf]

Supplementary Table S1. The effect of the ethanol or acetone concentration (%) and of UAE on the initial concentration (C_0) and the rate constant (k) of each extraction step of rosmarinic acid, total flavonoids and total phenolic diterpenes (sum of carnosic acid plus carnosol), as calculated by fitting Eq. 1. The correlation coefficients (R^2) are also presented.

	Rosmarinic acid					Flavonoids					Phenolic Diterpenes				
		(fast stage)		(slow stage)			(fast stage)		(slow stage)			(fast stage)		(slow stage)	
	C_0	k	R^2	k	R^2	C_0	k	R^2	k	R^2	C_0	k	R^2	k	R^2
**Ethanol concentration (%) in agitated reactor**															
**0**	7,01	0,023	0,99	0,012	0,99	2,51	0,004	0,99	0,001	0,93	n/a	n/a	n/a	n/a	n/a
**60**	3,27	0,018	0,99	0,006	0,98	1,59	0,015	0,99	0,010	0,99	14,73	0,026	0,94	0,008	0,97
**80**	2,28	0,014	0,99	0,005	0,99	5,67	0,008	0,99	0,003	0,99	20,45	0,027	0,99	0,009	0,99
**96**	0,81	0,004	0,98	0,001	0,84	3,76	0,002	0,99	0,001	0,99	19,73	0,025	0,97	0,007	0,99
**Acetone concentration (%) in agitated reactor**															
**0**	7,01	0,023	0,99	0,012	0,99	2,51	0,004	0,99	0,001	0,93	n/a	n/a	n/a	n/a	n/a
**40**	4,52	0,020	0,95	0,007	0,89	1,21	0,023	0,99	0,004	0,99	4,25	0,009	0,99	0,004	0,91
**60**	4,77	0,022	0,99	0,009	0,99	3,27	0,032	0,99	0,014	0,97	20,90	0,031	0,99	0,007	0,99
**80**	2,29	0,021	0,99	0,006	0,96	2,86	0,028	0,99	0,009	0,99	21,70	0,045	0,99	0,009	0,87
**100**	0,34	0,002	0,99	0,001	0,99	4,18	0,002	0,99	0,001	0,99	21,38	0,026	0,99	0,003	0,99
**Ultrasound Assisted Extraction**															
**60% EtOH**	4,52	0,054	0,99	0,020	0,98	4,34	0,063	0,98	0,020	0,99	22,80	0,066	0,99	0,043	0,99
**60% Acetone**	4,69	0,042	0,99	0,011	0,97	4,21	0,038	0,98	0,007	0,95	20,54	0,036	0,96	0,008	0,93
**80% Acetone**	7,35	0,025	0,94	0,009	0,99	4,96	0,029	0,97	0,005	0,99	23,37	0,048	0,99	0,020	0,99

*C_0 (mg/g_{dw})

*k (min^{-1})

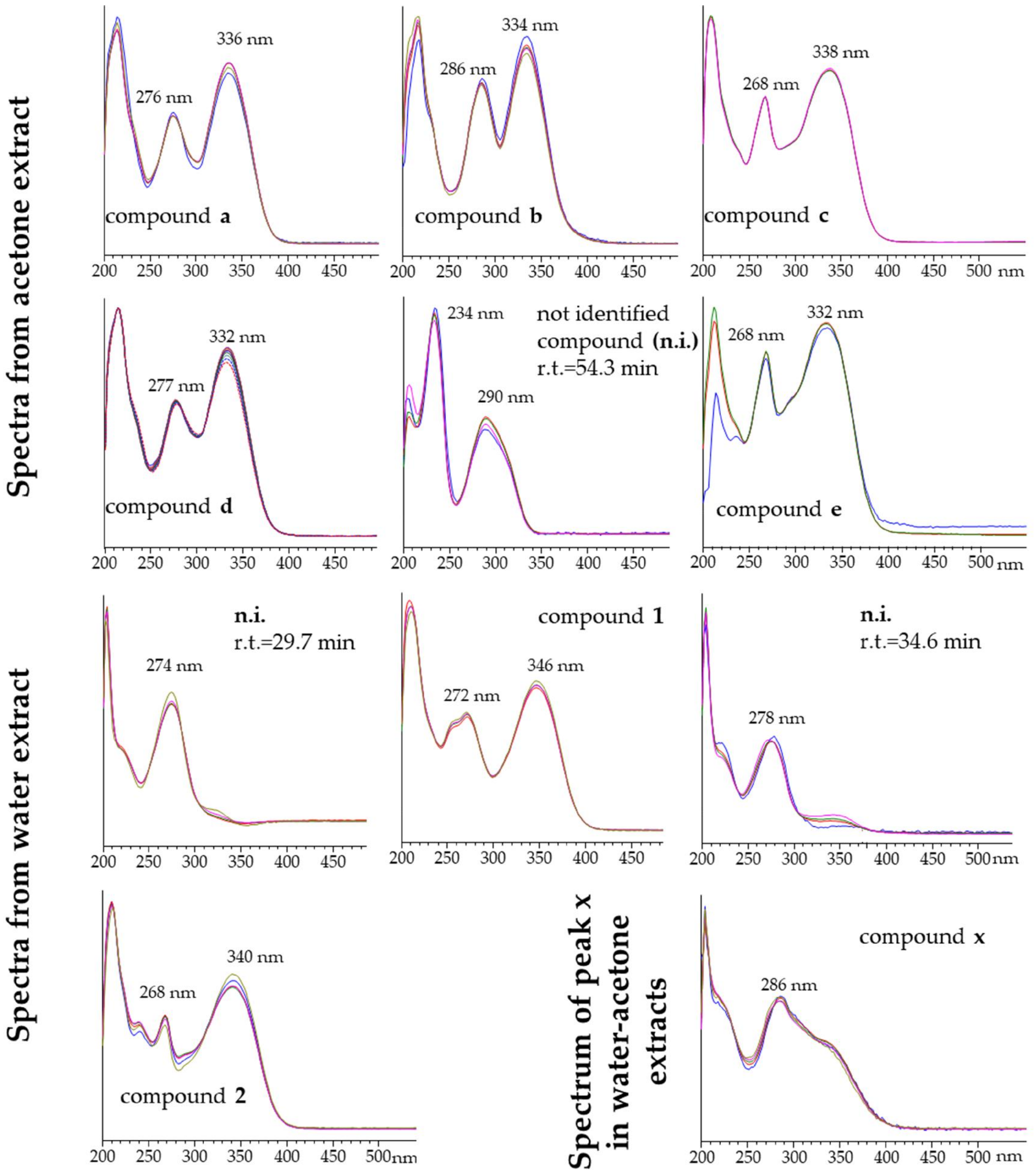

Supplementary Figure S1. The UV-Vis spectra of the minor peaks detected in the chromatograms of the water, acetone 100%, and water-acetone extracts.

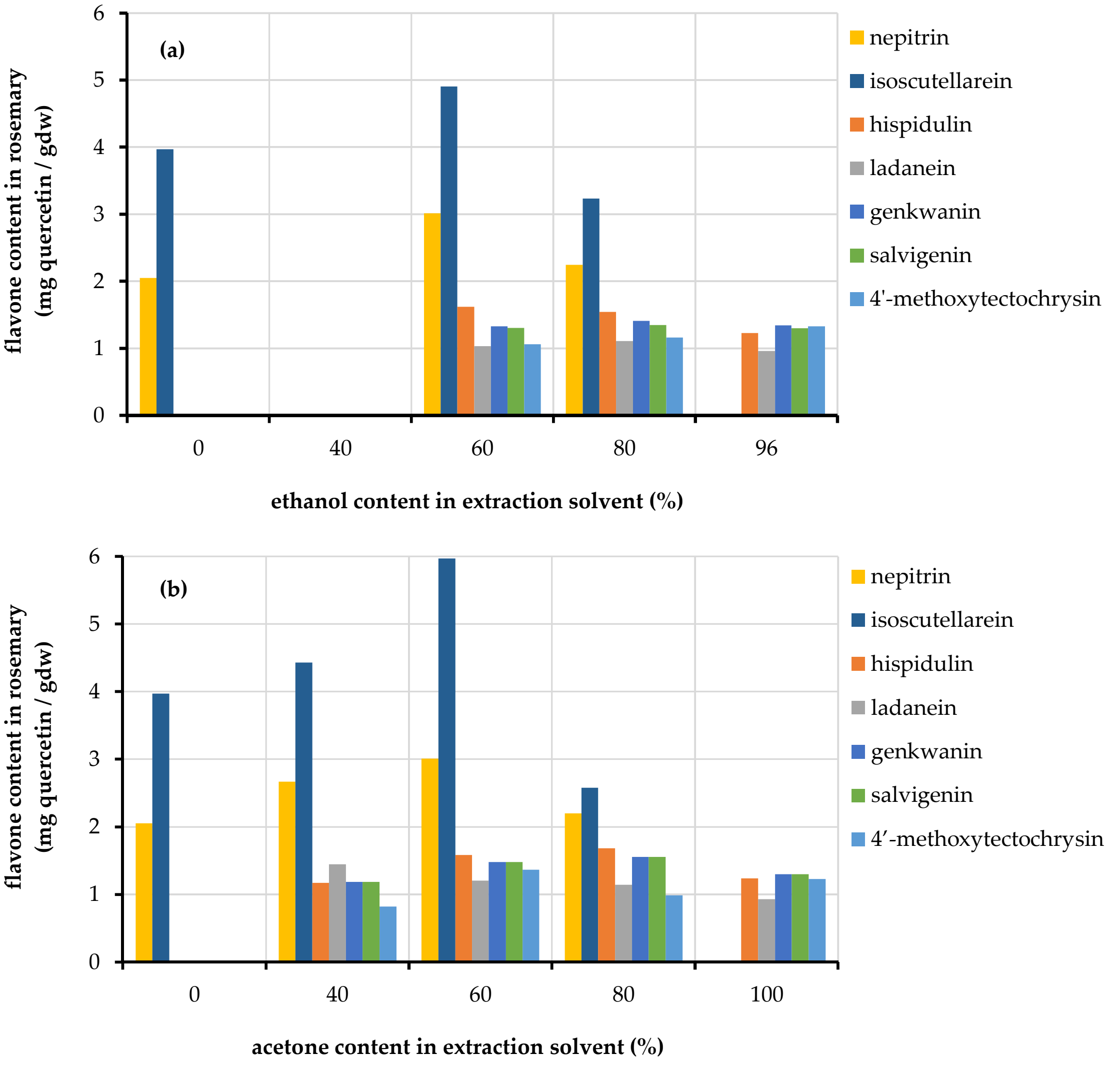

Supplementary Figure S2. The recovery of the individual identified flavones with the increase of ethanol (a) and acetone (b) in extraction solvent.
